# A Systematic Review and Meta-Analysis of Tai Chi Training in Cardiorespiratory Fitness of Elderly People

**DOI:** 10.1155/2022/4041612

**Published:** 2022-03-16

**Authors:** Tianyang Tan, Yanyan Meng, Jiaxuan L Lyu, Chaoyang Zhang, Chengchao Wang, Meng Liu, Xirui Zhao, Tianyi Lyu, Yulong Wei

**Affiliations:** ^1^School of Acupuncture-Moxibustion and Tuina, Beijing University of Chinese Medicine, Beijing 100029, China; ^2^Beijing University of Chinese Medicine, Beijing Research Institute of Chinese Medicine, Beijing 100029, China

## Abstract

**Objectives:**

The purpose of this study was to investigate the influence of Tai Chi on cardiorespiratory fitness (CRF) in elderly people using meta-analysis.

**Methods:**

This study used seven electronic databases and data retrieved from randomized controlled trials (RCTs) investigating the role of Tai Chi on CRF in the elderly. All these 24 RCTs were screened and selected from 7 literature databases. The Stata 11.2 software (StataCorp, USA) was used for the meta-analysis, subgroup analysis, and bias test, while the Cochrane Collaboration's tool was used for the assessment of the risk of bias (RoB). 4 researchers independently participated in sample selection, data extraction, and RoB assessment.

**Results:**

Following the inclusion criteria, 24 eligible studies were included in our analysis. The meta-analysis indicated that Tai Chi practice significantly increased the maximum rate of oxygen consumption (VO_2 max_) (weighted mean difference (WMD)  = 3.76, 95% CI: 1.25 to 6.26, *P* < 0.1), leading to an overall reduction in the heart rate (HR) (WMD  = −1.84, 95% CI: −2.04 to −1.63, *P*  ≤ 0.001) and an increase in the O_2 pulse_ (WMD = 0.94, 95% CI: 0.60 to 1.28, *P* ≤ 0.001) in individuals who practiced Tai Chi regularly compared with those who did not. The subgroup analysis suggested that overall in those who practiced Tai Chi, males (WMD = 1.48, 95% CI: 0.85 to 2.12, *P* ≤ 0.001) had higher O_2 pulse_ than females (WMD = 0.73, 95% CI: 0.33 to 1.12, *P* ≤ 0.001). The subgroup analysis also showed an increase in the vital capacity (VC) (WMD = 316.05, 95% CI: 239.74 to 392.35, *P* ≤ 0.001) in individuals practicing Tai Chi. When the samples were further stratified by Tai Chi practicing time, the subgroup analysis suggested that individuals practicing Tai Chi over a period of 24 weeks showed no significant difference in VC (WMD = 82.95, 95% CI: -98.34 to 264.23, *P*=0.370), while those practicing Tai Chi over a period of 48 weeks showed a significant increase (WMD = 416.62, 95% CI: 280.68 to 552.56, *P* ≤ 0.001). Furthermore, the subgroup analysis demonstrated that the increase in VC is significantly correlated with the Tai Chi practicing time (WMD = 344.97, 95% CI: 227.88 to 442.06, *P* ≤ 0.001).

**Conclusion:**

Regular Tai Chi practice could improve the CRF in the elderly, as indicated by significant improvement in indicators including VO_2__max_, O_2__pulse,_ VC, and HR. However, gender and practice time might influence the overall beneficial outcomes.

## 1. Introduction

Cardiorespiratory fitness (CRF) represents the capacity of the circulatory and respiratory systems to supply oxygen during sustained physical activity. Natural processes such as aging, senescence, and chronic diseases [1] often lead to an overall decline in the CRF [2], which is more pronounced in males than in females [3]. A positive correlation was observed between a steady decrease in CRF over time and an increase in the total mortality [4]. Maximal oxygen uptake (VO_2 max_), which decreases at an average rate of 1% per year after the age of 25, is a remarkable predictor of CRF [5]. High incidence of cardiovascular and respiratory diseases is particularly common in populations over the age of 45 [6]. Moreover, studies have found a direct correlation between poor CRF and increased risk of stroke (occurrence and recurrence) [7], atherosclerosis [8], type 2 diabetes [9], and disturbed cerebral blood flow (CBF), which can potentially impact brain structural and functional integrity and cognitive function [10].

Treatment options for cardiopulmonary rehabilitation include aerobic exercises [11], acupuncture [12], and the application of Chinese medicine [13]. However, the factors such as fear of needles and invasive therapeutic methods, and high medical expenses contributed to the avoidance of cardiopulmonary rehabilitation among patients. Aerobic exercise is widely recognized for its role in improving cardiac health and thus has always been recommended by doctors as a treatment option for cardiopulmonary rehabilitation aimed at prevention and recovery from preexisting diseases. Tai Chi involves slow-paced aerobic exercises with moderate intensity and combines delicate physical movements with rhythmic breathing [14], allowing adults of all age groups to participate. Thus, Tai Chi has gained popularity over the past years. In recent years, an increasing number of randomized control trials evaluating the beneficial effects of Tai Chi on balance function [15], fibromyalgia [16], and cognitive function have been carried out [17]. Some studies have reported the beneficial effects of Tai Chi on CRF in the elderly, while others have not, probably due to differences in geographic locations and practice intensity. To resolve the disparity in these studies, we conducted a systematic literature review and meta-analysis to elucidate the effects of Tai Chi on CRF in the elderly.

## 2. Materials and Methods

Our study design followed the guidelines for reporting systematic reviews in the Preferred Reporting Items for Systematic Reviews and Meta-Analyses (PRISMA) statement.

### 2.1. Literature Search Strategy

The protocol of this study was registered with the International Prospective Register of Systematic Reviews (PROSPERO) (registration number: CRD42021272968). The relevant literature studying the relationship between Tai Chi and cardiorespiratory fitness in elderly people was searched in 7 databases, including PubMed, Web of Science, EMBASE, Cochrane Library, Chinese Scientific Citation Database (CSCD), China National Knowledge Infrastructure Database (CNKI), and WanFang Database. The date of literature searching is from inception to June 9, 2021. Relevant systematic reviews and the reference list of included articles were searched to identify any further relevant studies.

The search keywords used in Chinese were as follows: “Tai Chi,” “cardiorespiratory function,” and “aged.” Based on similar studies [18], the search keywords in English used were used as follows (example from PubMed database quoted below):

  #1 Taiji [Mesh] OR Tai Chi [Title/Abstract] OR Chi, Tai [Title/Abstract] OR Tai Ji Quan [Title/Abstract] OR Ji Quan, Tai [Title/Abstract] OR Quan, Tai Ji [Title/Abstract] OR Taiji OR Taijiquan [Title/Abstract] OR T'ai Chi [Title/Abstract] OR Tai Chi Chuan [Title/Abstract].
  #2 Cardiorespiratory function [Mesh] OR maximal oxygen [Title/Abstract] OR FVC [Title/Abstract] OR Forced Vital Capacity [Title/Abstract] OR gas exchange rate [Title/Abstract] OR stroke volume [Title/Abstract] OR VE [Title/Abstract] OR minute ventilation [Title/Abstract] OR minute respiratory volume [Title/Abstract] OR EWK [Title/Abstract] OR myocardial oxygen consumption [Title/Abstract] OR HOV [Title/Abstract] OR MOCI [Title/Abstract] OR HOI [Title/Abstract] OR maximal oxygen consumption [Title/Abstract] OR FEK [Title/Abstract] OR expansion coefficient elasticity blood vessels [Title/Abstract] OR heart rate [Title/Abstract] OR blood pressure [Title/Abstract] OR oxygen pulse [Title/Abstract].
  #3 Aged [Mesh] OR elderly [Title/Abstract].
  #4 Control OR comparison OR controlled trial.
  #5 #1 AND #2 AND #3 AND #4.

### 2.2. Study Selection Criteria

The articles were primarily screened based on their titles and abstracts. Then, the full texts of these articles were further reviewed by 4 researchers. In the case of disagreement for study inclusion, the researchers would discuss until a consensus was reached. Studies were considered eligible if:

1. The mean age of patients was >50 years.
2. Tai Chi training was the sole intervention method irrespective of the style.
3. The outcomes included CRF parameters such as VO_2_, vital capacity (VC), and heart rate (HR).
4. Paired groups, including the control group (sedentary lifestyle) and the comparison group (practicing other forms of exercise such as walking or maintaining usual physical activity), were included in the study.
5. Language of publication was either English or Chinese.
6. The study was an RCT.

Studies were excluded if:

1. The study was a review, case study, or report describing a method or protocol.
2. The study cases were already included in another study we have selected.
3. Missing control groups or comparison groups.
4. Incomplete data.
5. The intervention group had a combinatorial exercise regime involving other forms of exercise training (e.g., strength training).

### 2.3. Data Extraction and Risk-of-Bias (RoB) Assessment

Two independent researchers participated in the data extraction. In case of disagreement, the researchers would discuss until a consensus was reached. The key data extracted from each study were as follows: (1) author details; (2) year of publication; (3) country; (4) sample size (M/F); (5) mean age (Tai Chi group/control group); (6) style of Tai Chi practiced; (7) frequency of exercise; (8) daily duration of exercise; (9) total time of Tai Chi training; and (10) the outcomes of CRF should include at least of the following core outcomes such as VO_2 max_ (mL kg^−1^ min^−1^, VC (mL), HR (beats per min), and O_2 pulse_ (mL beat^−1^).

The two researchers independently assessed the methodologies of the studies using the Cochrane Collaboration's tool for the assessment of RoB. The RoB assessment involved random sequence generation, allocation concealment, blinding of participants and personnel, blinding of outcome data, marking of incomplete outcome data, selective reporting, and screening of other existing biases.

### 2.4. Statistical Analysis

The Stata 11.2 software (StataCorp, USA) was used for conducting the meta-analysis. Regarding continuous variables, several analyses, such as combined effects, heterogeneity analysis, subgroup analysis, and publication bias analysis, were carried out. The calculated results were expressed as weighted mean difference (WMD). The *I*^*2*^ and *χ*^2^ homogeneity tests were conducted before the combined effects were evaluated. When *I*^*2*^ < 50% or *P* > 0.1, the variables were considered to possess low heterogeneity. When *I*^*2*^ < 50% or *P* < 0.1, the variables were considered to possess high heterogeneity. A fixed meta-analysis was performed when *I*^*2*^ < 50%, and a random meta-analysis was performed when *I*^*2*^ ≥50%. The publication bias analysis was conducted using Egger's and Begg's plots in the Stata 11.2, and the results are represented using a funnel chart.

## 3. Results

### 3.1. Study Selection

**Figure 1:**
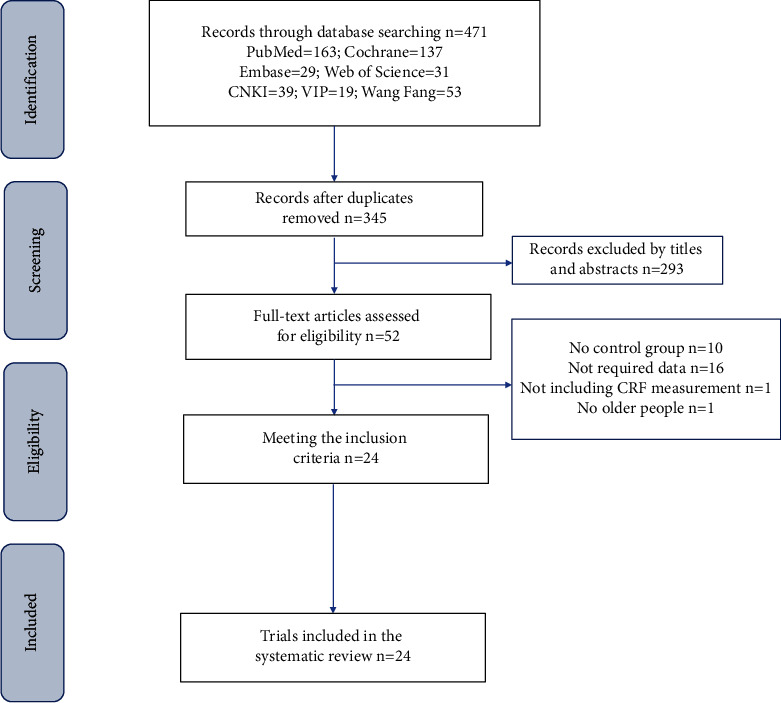
Flowchart representing the study selection criteria.

A total of 471 articles were identified from the search results of the 7 electronic databases. A total of 126 articles were excluded due to duplicate representation (Figure 1). After reading the titles and abstracts, we rescreened the remaining 52 articles. We excluded 10 articles due to lack of inclusion of control group; 16 articles due to the presence of unrelated data not pertinent to this study; 1 article due to inclusion of non-elderly; and 1 article due to missing of information on CRF measurements. Finally, a total of 24 articles that met the eligibility criteria for the systematic review were included.

### 3.2. Characteristics of Selected Studies

The 24 articles selected for the meta-analysis reported data from 1995 to 2020 and represented individuals geographically localized in countries such as China, the Netherlands, Mexico, and the United States. The sample size of these studies ranged from 20 to 380. A total of 2155 participants were included, with ages ranging from 50 to 89. The most common frequency of Tai Chi training reported was 4 times per week (20%). The longest duration of Tai Chi practice reported was 11 years, and the shortest was 4 weeks (Table 1).

### 3.3. RoB Results

**Figure 2:**
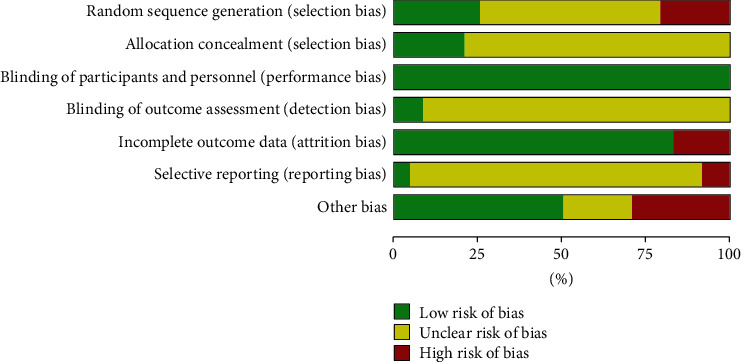
Graph representing the risk of bias (RoB).

**Figure 3:**
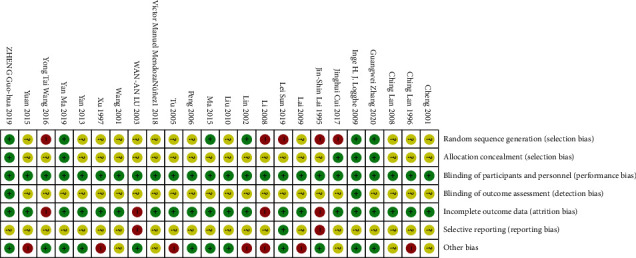
Summary of the risk of bias (RoB).

The results of the RoB of these RCTs are summarized in Figures 2 and 3, respectively. In summary, 6 studies (25%) showed low-risk bias due to random sequence generation; 5 studies (20%) showed low-risk bias attributed to allocation concealment; 24 studies (100%) exhibited low-risk bias due to blinding of the participants or personnel; 2 studies (8%) showed low-risk bias, which was attributed to blinding of the assessment outcomes; 20 studies (80%) showed a low-risk bias due to incompleteness of the outcome data; 1 study (4%) showed low-risk bias due to selective reporting; and 12 studies (48%) showed low-risk bias due to the presence of other factors or biases.

### 3.4. Meta-Analysis

#### 3.4.1. Tai Chi for VO_2 max_

**Figure 4:**
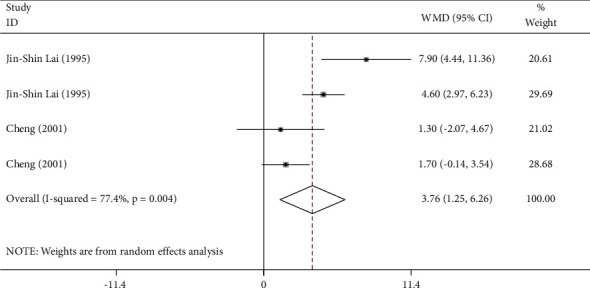
Forest plot representing the effect of Tai Chi on the VO_2 max_.

Two studies including 122 patients contributed to the meta-analysis of the VO_2 max_ (Figure 4). Tai Chi training significantly increased the VO_2 max_ compared with the control (WMD = 3.76, 95% CI: 1.25 to 6.26, *P*=0.003). The *I*^2^ was 77.4%, heterogeneity *χ*^2^ = 13.26 (d.f. = 3), and *P*=0.004. There was substantial heterogeneity across the studies included in the meta-analysis.

#### 3.4.2. Tai Chi for HR

**Figure 5:**
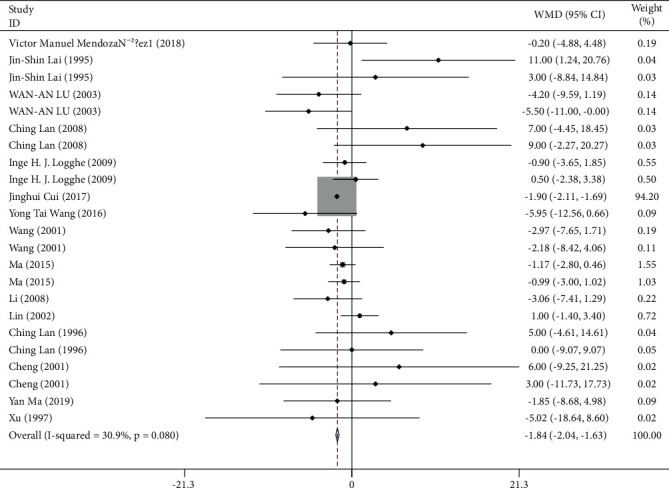
Forest plot representing the effect of Tai Chi on HR.

**Figure 6:**
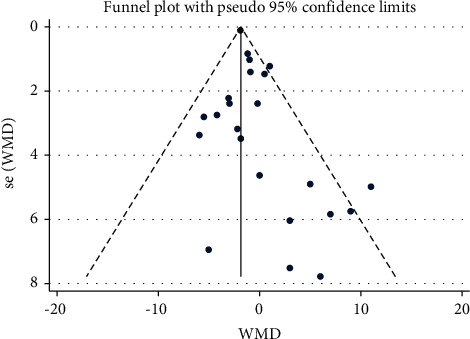
Funnel plot representing the effect of Tai Chi on HR.

1,492 participants from 15 studies were used for the meta-analysis of HR. As shown in Figure 5, the HR was significantly reduced in participants who practiced Tai Chi compared with those who did not (WMD = -1.84, 95% CI: -2.04 to -1.63, *P*=0.001). For these studies, I^2^ = 30.9%, *χ*^2^ = 31.83 (d.f. = 22), and *P*=0.008, indicating a low heterogeneity across these studies. The funnel plots for several outcomes were not fully symmetrical (Figure 6). The *P* value for Egger's test was 0.026. The *Z* value for Begg's test was 0.79 (Figure 6).

#### 3.4.3. Tai Chi for O_2 pulse_

**Figure 7:**
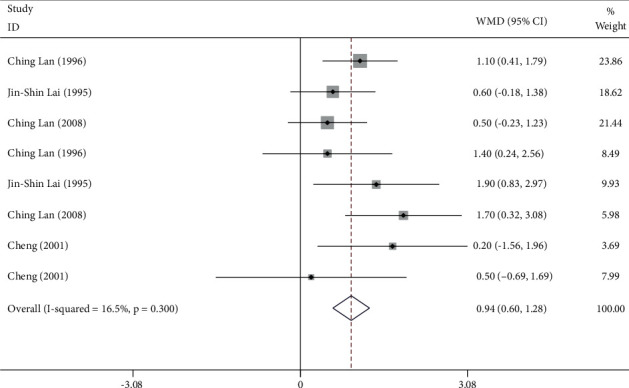
Forest plot representing the effect of Tai Chi on O_2 pulse_.

267 participants from 4 studies were used for the meta-analysis of O_2 pulse_. As shown in Figure 7, the O_2 pulse_ was significantly increased in participants who practiced Tai Chi compared with those who did not (WMD = 0.94, 95% CI: 0.60 to 1.28, *P*=0.001). For these studies, *I*^2^ = 16.5%, *χ*^2^ = 8.38 (d.f. = 7), and *P*=0.300, indicating a low heterogeneity across these studies.

**Figure 8:**
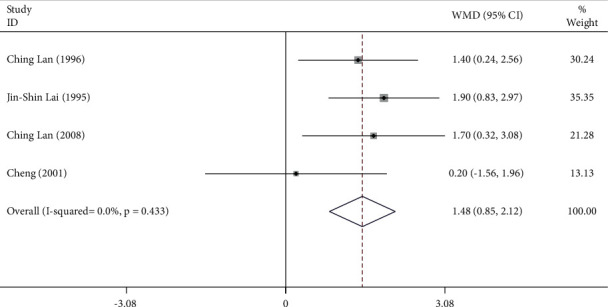
Forest plot representing the effect of Tai Chi on O_2 pulse_ in males.

**Figure 9:**
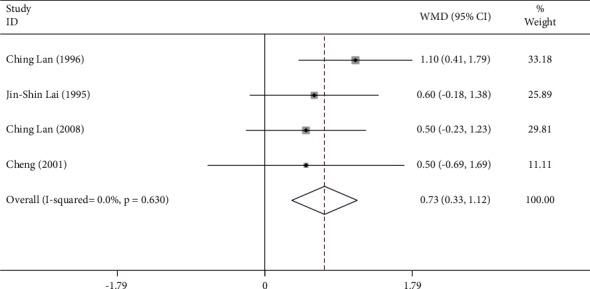
Forest plot representing the effect of Tai Chi on O_2 pulse_ in females.

The subgroup analysis was performed to compare the effects of Tai Chi across different genders in the test population. The results suggested that Tai Chi practice in males resulted in a significant increase in the O_2 pulse_ (WMD = 1.48, 95% CI: 0.85 to 2.12, *P*=0.001) (Figure 8). No heterogeneity was observed in these studies that included male participants, as indicated by *I*^2^ = 0.0% (Figure 8). Similarly, Tai Chi practice in females significantly increased the O_2 pulse_ as well (WMD = 0.73, 95% CI: 0.33 to 1.12, *P*=0.001) (Figure 9). No heterogeneity was observed in these studies that included female participants (*I*^2^ = 0.0%) (Figure 9)

#### 3.4.4. Tai Chi for VC

**Figure 10:**
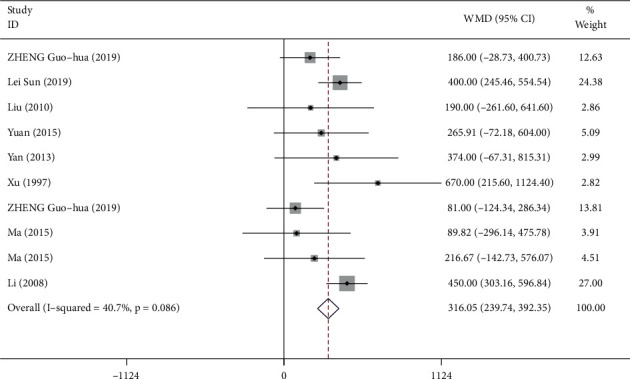
Forest plot representing the effect of Tai Chi on VC.

**Figure 11:**
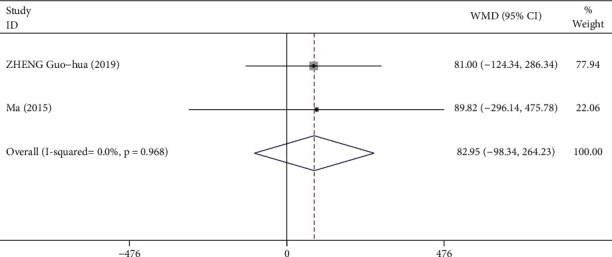
Forest plot representing the effect of Tai Chi on VC for individuals undergoing Tai Chi training for a period of 24 weeks.

748 participants from 8 studies were used for the meta-analysis of VC. As shown in Figure 10, the VC was significantly increased in participants who practiced Tai Chi compared with those who did not (WMD = 3 16.05, 95% CI: 239.74 to 392.35, *P*=0.001). For these studies, the *I*^2^ = 40.7%, heterogeneity *χ*^2^ = 5.17 (d.f. = 9), and *P*=0.086, indicating a low heterogeneity across the studies. The funnel plots for several outcomes were not fully symmetrical (Figure 11). *P* value for Egger's test was *P*=0.464, and *Z* value for Begg's test was 0.09.

**Figure 12:**
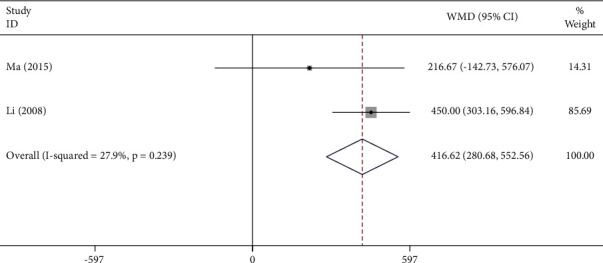
Forest plot representing the effect of Tai Chi on VC for individuals undergoing Tai Chi training for a period of 48 weeks.

The subgroup analysis was performed to compare the effects of Tai Chi practice of different exercise durations (Figure 12). The results suggested that no statistically significant difference existed between the control and comparison groups with participants undergoing Tai Chi training less than 24 weeks (WMD = 82.95, 95% CI: -98.34 to 264.23, *P*=0.370).

**Figure 13:**
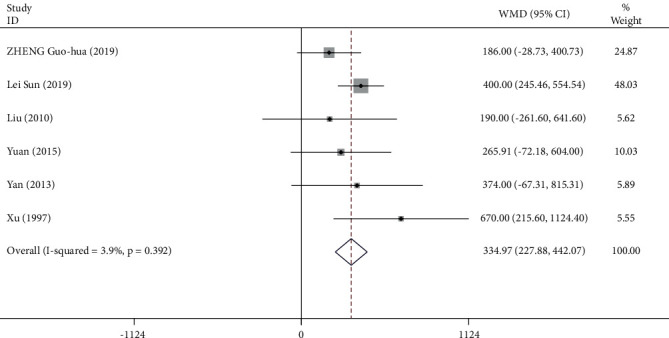
Forest plot representing the effect of Tai Chi on VC for individuals undergoing the Tai Chi training for mixed durations.

The VC was increased in the Tai Chi practice group with a duration of 48 weeks compared with that of the control group (WMD = 416.62, 95% CI: 280.68 to 552.56, *P* ≤ 0.001). In these studies, *I*^2^ = 27.9%, indicating a low heterogeneity across the studies (Figure 13).

**Figure 14:**
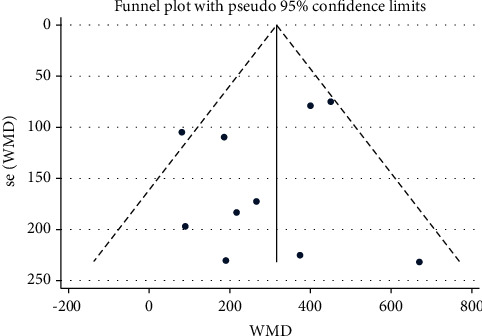
Funnel plot representing the effect of Tai Chi on VC.

The Tai Chi training for mixed duration significantly increased the VC compared with the control (WMD = 344.97, 95% CI: 227.88 to 442.06, *P* ≤ 0.001). There was no heterogeneity across the studies included in the meta-analysis (*I*^2^ = 3.9%) (Figure 14).

## 4. Discussion

Tai Chi is originated from traditional Chinese martial arts and medicine [43] and was practiced to maintain physical and mental health. The unity of opposites representing yin-yang is also an integral part of the symbol representing Tai Chi. Tai Chi is also known as the “moving meditation” [44]. This study aimed to evaluate the effectiveness of Tai Chi in improving CRF in the elderly using a meta-analysis approach, which included 2155 participants from 24 RCTs. Based on our analyses, we concluded that overall the Tai Chi training could significantly improve the CRF in the elderly. However, the beneficial effects of Tai Chi are influenced by many factors, including gender and practice time.

VO_2 max_ and O_2 pulse_ were indicators of comprehensive circulatory and respiratory ability; in particular, VO_2 max_ was the gold parameters of CRF. The parameters of cardiorespiratory fitness are various, such as maximal minute ventilation (MMV) and cardio output (CO). However, there were very few literatures including MVV and CO. Thus, we were unable to perform meta-analysis. Blood pressure was a vital sign, and the change in blood pressure was not influenced by a single factor of Tai Chi training. Therefore, blood pressure was not selected as a CRF indicator in this study.

### 4.1. VO_2 max_

The VO_2 max_ represents the oxygen consumed during a maximum intensity exercise, which can be analyzed using a cardiopulmonary exercise test (CEPT). The VO_2 max_ is an indicator of CRF [45]. The results of the meta-analysis suggested that Tai Chi training could significantly improve the VO_2 max_ in individuals who practiced Tai Chi compared with those who did not (WMD = 3.76, 95% CI: 1.25 to 6.26, *P*=0.003). The improvement of VO_2 max_ might be attributed to the distinct movement patterns performed during the practice of Tai Chi. Tai Chi training requires the center of gravity to move down, the waist to rotate slowly, and the upper and lower limbs to coordinate simultaneously. The overall rhythm of Tai Chi training is regular, involving movements of the abdominal muscles, pectoralis major, and sternocleidomastoid muscle (breathing muscles). The Tai Chi movements also involve trained and rhythmical breathing. The fusion of body exercise and effective breathing enhances the contractility and endurance of the diaphragm.

Although VO_2 max_ is currently the “gold standard” for CRF, there are very few published articles that used CEPT to evaluate the efficacy of Tai Chi. This may be due to the fact that the presence of preexisting chronic diseases in the elderly hindered the performance of CEPT, as this test method involves high-risk events such as palpitations, loss of consciousness, and, more seriously, a sudden death.

### 4.2. HR

HR is a commonly measured vital sign, which is regulated by the autonomic nervous system [46]. Our analyses showed that the Tai Chi training significantly reduced the HR in those who practiced Tai Chi (WMD = −1.84, 95% CI: −2.04 to −1.63, *P* ≤ 0.001). A previous study has proved that Tai Chi could enhance parasympathetic activity and decrease sympathetic activity [47]. The neurophysiological mechanism of Tai Chi may involve the activation of the parasympathetic nervous system, which is known to decrease HR [48] and play an active role in relieving anxiety and fear [49]. Elevated HR can increase the risk of sudden death [50]. Based on these studies, we believe that long-term and regular Tai Chi training could extend the life span of the elderly and increase the happiness index of life for them. According to Begg's test (*P*=0.026) and the funnel plot, there is a significant bias in the published studies, probably due to the fact that only positive results are likely to be published. Nevertheless, we suppose that the results accurately represent the real-life situation, as a recently published study report confirmed the results [51].

### 4.3. O_2 pulse_

The O_2 pulse_ reflects the oxygen intake per heartbeat and is represented by the ratio of oxygen consumption to HR. The increase in O_2 pulse_ indicates superior cardiopulmonary fitness during exercise. Our results suggested that Tai Chi could improve the O_2 pulse_ in those who practiced Tai Chi (WMD = 0.94, 95% CI: 0.60 to 1.28, *P* ≤ 0.001). The subgroup analysis suggested that males (WMD = 1.48, 95% CI: 0.85 to 2.12, *P* ≤ 0.001) had higher O_2 pulse_ than females (WMD = 0.73, 95% CI: 0.33 to 1.12, *P* ≤ 0.001). Females, on average, have smaller body sizes and organs, while their body fat is usually higher than males [52]. Additionally, females seem to be more vulnerable to cardiovascular diseases caused by obesity [53]. High body fat is a negative factor [54] limiting the performance of females during Tai Chi training. Also, for participants who practiced Tai Chi, males have better CRF than females, a difference estimated to be 20% [55], which is consistent with our findings. The average O_2 pulse_ in females is less than what is observed in males. There was limited literature in this context, preventing us from drawing more precise conclusions.

Our results also suggested that gender could affect the effectiveness of Tai Chi in individuals. After Tai Chi-based interventions were initiated, males showed higher O_2 pulse_ than females. The factors such as gender, body fat, and fat metabolism should be incorporated to develop a more personalized Tai Chi training regime in rehabilitation clinics.

### 4.4. VC

The VC is one of the most commonly used indicators for evaluating the physiologic and pathophysiologic state of the lungs, due to the simple and fast measurement process. VC is an indicator for the inspiratory reserve capacity, expiratory reserve capacity, and tidal volume of an individual [56]. VC is also used for the diagnosis of lung diseases such as chronic obstructive pulmonary disease (COPD) [57] and asthma [58]. Our results suggested that Tai Chi significantly increased the VC in individuals (WMD = 316.05, 95% CI: 239.74 to 392.35, *P* ≤ 0.001). In 8 studies for individuals with a short practice duration of 4 weeks, the WMD was 670.00 with a 95% CI ranging from 215.60 to 1124.40. In individuals who underwent a longer training duration (96 weeks), the WMD was 265.92, with a 95% CI ranging from 72.18 to 604.00. This sharp increase in VC may be associated with a cardiorespiratory stress reaction to short periods of high-frequency Tai Chi-based training (7 times per week, 60 min per day, and a total duration of 4 weeks). When the training lasted 96 weeks, the beneficial effect on the VC may not be evident. Overtraining often reduces skeletal muscle strength [59] and induces oxidative stress [60], which may trigger a ceiling effect. According to our subgroup analysis, VC was higher in individuals who underwent Tai Chi training for 48 consecutive weeks (WMD = 416.62, 95% CI: 280.68 to 552.56, *P* ≤ 0.001) than those who practiced Tai Chi for 24 consecutive weeks (WMD = 82.95, 95% CI: -98.34 to 264.23, *P*=0.370). The increase in VC for individuals practicing Tai Chi for 24 weeks was reversed when the training was done over a 48-week period. We hypothesized that the improvement of VC could only be achieved by compounding the effects of exercise over a longer training duration. However, VC turned out to be an indicator with reduced sensitivity, which changed significantly with long durations of Tai Chi training. Tai Chi involves aerobic exercises of low-to-moderate intensity. Determining the training duration may provide better clinically significant insight. According to Begg's test (*P*=0.464) and the funnel plot, there was no bias in the published studies.

## 5. Conclusions

To the best of our knowledge, our study is the first to report the correlation between CRF and Tai Chi training in the elderly using meta-analysis. Our findings suggest that Tai Chi training effectively improved the CRF in older adults. We demonstrated that practicing Tai Chi could benefit body function by enhancing factors such as VO_2 max_, O_2 pulse_, VC, and HR. Additionally, we found that gender and practice time can also influence the outcome of Tai Chi practice. Compared with females, males may benefit to a greater extent showing better CRF. We also demonstrated that longer practice time could improve the CRF. Thus, this study contributes to the existing knowledge and provides a new direction for further study.

### 5.1. Limitations

There were several limitations of this study. (1) Although we included studies from multiple databases, we only considered studies published in English and Chinese, which might undergo the risk of miss studies; (2) limiting the number of studies by our inclusion criteria may lead to bias; and (3). The quality of included literature was low methodological. The descriptions of the 18 studies regarding the random sequence generation were not detailed. There were no descriptions of allocation concealment in 19 studies. 22 studies have the risk in blinding of the assessment outcomes. These limitations could possibly attribute to multiple factors. First, how Tai Chi could relate to CRF has not attracted enough attention. Second, Tai Chi as the exercise therapy was unable to be blinded. Lastly, Tai Chi training requires disciple and it is rather difficult to adhere to a regular training regimen. (4) The subgroup analysis of the control group was not performed, which may further contribute to biased results. Therefore, additional RCTs with larger sample sizes would be essential in future studies.

## Figures and Tables

**Table 1:** Details of studies that were included in the meta-analysis.

Author, year	Country and languages	Sample size (M/F)	Mean age (T/C)	Control group	Tai Chi style	Frequency	Daily time	Duration	Outcome
Lan et al., 1996 [19]	China English	76 40/36	69.3 ± 3.9	NC	Classical Yang's Tai Chi	4.3 ± 1.3 time per week	20 min of warm up, 24 min Tai Chi, 10 min of cool down	11.8 ± 5.6 years	HR, O_2 pulse_
Zheng et al., 2019 [20]	China English	170 52/118	61.01 ± 5.20/60.73 ± 6.05	Physical activities	24-movement Yang-style Tai Chi	5 time per week	10 min of warm up, 45 min Tai Chi, 5 min of cool down	12 weeks	VC
Lu and Kuo, 2003 [21]	China English	40 14/26	56.3 ± 8.5/52.8 ± 7.5	No physical exercise	Classical Yang's Tai Chi	NC	10 min of warm up, 20 min Tai Chi, 10 min of cool down	NC	HR
Mendoza-Núñez1 et al., 2018 [22]	Mexico English	85	68.2 ± 6.6/67.4 ± 4.7	No physical exercise	Eight-form easy Tai Chi for elderly adults	5 time per week	10 min of warm up, 30 min Tai Chi, 5 min of cool down	24 weeks	HR
Sun et al., 2019 [23]	China English	120 68/52	65.2 ± 9.2/66.4 ± 10.0	Activities of daily living	24-movement Yang-style Tai Chi	3 times per week	30–40 min	NC	VC
Lai et al., 1995 [24]	China English	84 44/40	64 ± 9	NC	Classical Yang's Tai Chi	5.0 ± 1.1times per week	20 min of warm up, 24 min Tai Chi, 10 min of cool down	24 weeks	HR, O_2 pulse_, VO_2 max_
Lan et al., 2008 [25]	China English	69 34/35	64.0 ± 6.8/64.7 ± 7.4	No physical exercise	Classical Yang's Tai Chi	NC	20 min of warm up, 24 min Tai Chi, 10 min of cool down	240 weeks	HR, O_2 pulse_
Logghe et al., 2009 [26]	NED English	269 78/191	76.8 ± 4.6/77.5 ± 4.7	Usual care	10-movement Yang-style Tai Chi	2 times per week	1 hour	13 weeks	HR
Ma e t al., 2019 [27]	US English	52 18/34	64.85 ± 7.62/64.15 ± 7.69	Usual care	NC	2 times per week	NC	24 weeks	HR
Cui and Fu, 2017 [28]	China English	140	68.4 ± 3.2	Slow walking exercise	NC	4 times per week	30–60 min	24 weeks	HR
Wang et al., 2016 [29]	US English	28 7/21	89.73 ± 6.31/87.23 ± 6.71	NC	10-form Tai Chi	2 times per week	10 min of warm up, 45 min Tai Chi, 5 min of cool down	12 weeks	HR
Zhang et al., 2020 [30]	China English	36	59.65 ± 8.42/62.21 ± 7.76	Physical activities	NC	NC	NC	12 weeks	HR
Wang et al., 2001[31]	China Chinese	115 60/55	68.81 ± 5.72/67.07 ± 4.98	No physical exercise	NC	NC	NC	NC	HR
Ma, 2015 [32]	China Chinese	27	60.86 ± 2.54/60.43 ± 1.90	No physical exercise	NC	NC	NC	24/48 weeks	VC, HR
Liu and Jin, 2010 [33]	China Chinese	20 10/10	61.7 ± 4.3	No physical exercise	24-movement Yang-style Tai Chi	4 times per week	10 min of warm up, 40 min Tai Chi, 10 min of cool down	8 weeks	VC
Li, 2008 [34]	China Chinese	60	66.1 ± 4.6/65.3 ± 4.8	No physical exercise	NC	4 times per week	40–60 min	48 weeks	HR
Peng, 2006 [35]	China Chinese	380 180/200	NC	No physical exercise	NC	3 times per week	30 min	240 weeks	VC
Yuan, 2015 [36]	China Chinese	100	61.18 ± 8.916/61.26 ± 8.813	No physical exercise	NC	3 times per week	30 min	96 weeks	VC
Yan, 2013 [37]	China Chinese	47 24/23	＞65	No physical exercise	24-movement Tai Chi	NC	30 min	NC	VC
Lai et al., 2009 [38]	China Chinese	64	68.4 ± 2.1/67.9 ± 2.4	Running training	NC	NC	NC	48 weeks	HR
Xu and Wen, 1997 [39]	China Chinese	34 17/17	64. 6 ± 3. 9/66. 7 ± 7. 4	No physical exercise	Yang-style Tai Chi	7 times per week	60 min	4 weeks	VC, HR
Tu, 2005 [40]	China Chinese	32	NC	Running training	NC	3 times per week	12 min	10 weeks	HR
Lin and Huang, 2002 [41]	China Chinese	69	50–62	Aerobic exercise	24-movement Yang-style Tai Chi	4 times per week	40 min	24 weeks	HR
Cheng et al., 2001 [42]	China Chinese	38 18/20	58–70	NC	Classical Yang's Tai Chi	4.6 ± 1.3 times per week	20 min of warm up, 24 min Tai Chi, 10 min of cool down	NC	HR, VO_2 max_, O_2 pulse_

## Data Availability

The data for supporting this review were taken from previously reported and datasets, which have been cited. Data are available upon request to the corresponding author.

## References

[1] Katzel L. I., Sorkin J. D., Fleg J. L. (2001). A comparison of longitudinal changes in aerobic fitness in older endurance athletes and sedentary men. *Journal of the American Geriatrics Society*.

[2] Zeiher J., Ombrellaro K. J., Perumal N., Keil T., Mensink G. B. M., Finger J. D. (2019). Correlates and determinants of cardiorespiratory fitness in adults: a systematic review. *Sports Medicine - Open*.

[3] Parker B. A., Kalasky M. J., Proctor D. N. (2010). Evidence for sex differences in cardiovascular aging and adaptive responses to physical activity. *European Journal of Applied Physiology*.

[4] Laukkanen J. A., Zaccardi F., Khan H., Kurl S., Jae S. Y., Rauramaa R. (2016). Long-term change in cardiorespiratory fitness and all-cause mortality. *Mayo Clinic Proceedings*.

[5] Mundwiler J., Schüpbach U., Dieterle T. (2017). Association of occupational and leisure-time physical activity with aerobic capacity in a working population. *PLoS One*.

[6] Sui X., LaMonte M. J., Laditka J. N. (2007). Cardiorespiratory fitness and adiposity as mortality predictors in older adults. *JAMA*.

[7] Hooker S. P., Sui X., Colabianchi N. (2008). Cardiorespiratory fitness as a predictor of fatal and nonfatal stroke in asymptomatic women and men. *Stroke*.

[8] Kim D., Park W. (2017). The inverse relationship between cardiorespiratory fitness and intima-media thickness with prehypertensive middle-aged women. *Tohoku Journal of Experimental Medicine*.

[9] LaMonte M. J., Barlow C. E., Jurca R., Kampert J. B., Church T. S., Blair S. N. (2005). Cardiorespiratory fitness is inversely associated with the incidence of metabolic syndrome. *Circulation*.

[10] Silvestre O. M., Nadruz W., Claggett G. (2018). Declining lung function and cardiovascular risk. *Journal of the American College of Cardiology*.

[11] Martens C. R., Kirkman D. L., Edwards D. G. (2016). The vascular endothelium in chronic kidney disease. *Exercise and Sport Sciences Reviews*.

[12] Ma W., Li Z., Lu Z. (2017). Protective effects of acupuncture in cardiopulmonary bypass-induced lung injury in rats. *Inflammation*.

[13] Zhou N., Sun Y. P., Zheng X. K. (2017). A metabolomics-based strategy for the mechanism exploration of traditional Chinese medicine: *Descurainia sophia* seeds extract and fractions as a case study. *Evidence-based Complementary and Alternative Medicine*.

[14] Miller S., Taylor-Piliae R. E. (2018). The association between Tai Chi exercise and safe driving performance among older adults: an observational study. *Journal of Sport and Health Science*.

[15] Li F., Harmer P., Fitzgerald K. (2012). Tai Chi and postural stability in patients with Parkinson’s disease. *New England Journal of Medicine*.

[16] Wang C., Schmid C. H., Fielding R. A. (2018). Effect of Tai Chi versus aerobic exercise for fibromyalgia: comparative effectiveness randomized controlled trial. *BMJ*.

[17] Wayne P. M., Walsh J. N., Taylor-Piliae R. E. (2014). Effect of Tai Chi on cognitive performance in older adults: systematic review and meta-analysis. *Journal of the American Geriatrics Society*.

[18] Zheng G., Li S., Huang M., Liu F., Tao J., Chen L. (2015). The effect of Tai Chi training on cardiorespiratory fitness in healthy adults: a systematic review and meta-analysis. *PLoS One*.

[19] Lan C., Lai J.-S., Wong M.-K., Yu M.-L. (1996). Cardiorespiratory function, flexibility, and body composition among geriatric Tai Chi Chuan practitioners. *Archives of Physical Medicine and Rehabilitation*.

[20] Zheng G.-h., Zheng X., Li J.-z., Duan T.-j., Tao J., Chen L.-d. (2019). Effect of Tai Chi on cardiac and static pulmonary function in older community-dwelling adults at risk of ischemic stroke: a randomized controlled trial. *Chinese Journal of Integrative Medicine*.

[21] Lu W. A., Kuo C. D. (2003). The effect of Tai Chi Chuan on the autonomic nervous modulation in older persons. *Medicine and Science in Sports and Exercise*.

[22] Mendoza-Núñez V. M., Arista-Ugalde T. L., Rosado-Pérez J., Ruiz-Ramos M., Santiago-Osorio E. (2018). Hypoglycemic and antioxidant effect of Tai Chi exercise training in older adults with metabolic syndrome. *Clinical Interventions in Aging*.

[23] Sun L., Zhuang L.-P., Li X.-Z., Zheng J., Wu W.-F. (2019). Tai Chi can prevent cardiovascular disease and improve cardiopulmonary function of adults with obesity aged 50 years and older. *Medicine*.

[24] Lai J.-S., Lan C., Wong M.-K., Teng S.-H. (1995). Two-year trends in cardiorespiratory function among older Tai Chi Chuan practitioners and sedentary subjects. *Journal of the American Geriatrics Society*.

[25] Lan C., Chen S. Y., Lai J. S. (2008). Changes of aerobic capacity, fat ratio and flexibility in older TCC practitioners: a five-year follow-up. *The American Journal of Chinese Medicine*.

[26] Logghe I. H. J., Zeeuwe P. E. M., Verhagen A. P. (2009). Lack of effect of Tai Chi Chuan in preventing falls in elderly people living at home: a randomized clinical trial. *Journal of the American Geriatrics Society*.

[27] Ma Y., Wu C.-w., Peng C.-K. (2019). Complexity-based measures of heart rate dynamics in older adults following long- and short-term Tai Chi training: cross-sectional and randomized trial studies. *Scientific Reports*.

[28] Cui J., Fu L. (2017). Effect of Taijiquan and slow walking on Chinese elderly female’s cardiovascular function and quality of life. *Biomedical Research*.

[29] Wang Y. T., Li Z., Yang Y. (2016). Effects of wheelchair Tai Chi on physical and mental health among elderly with disability. *Research in Sports Medicine*.

[30] Zhang G., Wang S., Gu Y., Song L., Yu S., Feng X. (2020). Tai Chi improves coronary heart disease risk by inactivating MAPK/ERK pathway through serum miR-126. *Evidence-based Complementary and Alternative Medicine: eCAM*.

[31] Wang W., Shen Y., Huang M. (2001). A comparative study on the effects of different exercise programs on cardiopulmonary function in the elderly. *Journal of Chengdu Physical Education Institute*.

[32] Ma Z. J. (2015). Effect of exercise intervention on dyslipidemia elderly. *Sport Science and Technology*.

[33] Liu X., Jin H. (2010). Observation on effect of Tai Chi chuan on cardiorespiratory function of older people. *China Practical Medical*.

[34] Li X. (2008). Effects of TaiChi exercise on cardiopulmonary function in elderly men. *Journal of Henan Normal University (Natural Science)*.

[35] Peng C. (2006). Effects of Tai Chi on body composition and cardiopulmonary function in the elderly. *Martial Arts Science*.

[36] Yuan Y. (2015). The role of Taijiquan on the elderly cardiopulmonary function and heart and cerebral vessels. *Zhong Zhou TiYu·Shao Lin and Tai Ji*.

[37] Yan Y. (2013). Study on the effect of 24 types of Tai Chi exercise on cardiopulmonary function in middle-aged and elderly people. *Journal of Liaoning Normal University (Natural Science Edition)*.

[38] Lai A., Hua M., Chen J. (2009). Discussion on different physical exercise on the mental health, cardiac and ventilatory function effect of the aged. *Zhejiang Sport Science*.

[39] Xu Z., Wen M. (1997). Changes of cardiopulmonary functions of senior people before and after taijiquan exercises. *Journal of Chengdu Physical Education Institute*.

[40] Tu H. L. (2005). Influences of different sports events on old people’s cardiovascular system. *Journal of Wuhan Institute of Physical Education*.

[41] Lin J., Huang C. (2002). A comparative study on the effect of aerobics and Taijiquan on the fitness of middle-aged and old women. *Journal of Shanghai Institute of Physical Education*.

[42] Cheng L., Jin S., Chen S., Wang M. (2001). Effects of 12 months of Tai Chi practice on health adaptability of the elderly. *Sports Technology Information*.

[43] Si Y., Wang C., Yin H. (2020). Tai Chi Chuan for subjective sleep quality: a systematic review and meta-analysis of randomized controlled trials. *Evidence-based Complementary and Alternative Medicine: eCAM*.

[44] Hawkes T. D., Manselle W., Woollacott M. H. (2014). Cross-Sectional comparison of executive attention function in normally aging long-TermT’ai Chi, meditation, and aerobic fitness practitioners versus sedentary adults. *Journal of Alternative & Complementary Medicine*.

[45] Edwards L. M., Kemp G. J., Dwyer R. M. (2013). Integrating muscle cell biochemistry and whole-body physiology in humans:31P-MRS data from the InSight trial. *Scientific Reports*.

[46] Zhu Z., Wang X., Li X. (2019). Genetic overlap of chronic obstructive pulmonary disease and cardiovascular disease-related traits: a large-scale genome-wide cross-trait analysis. *Respiratory Research*.

[47] Qi Y., Xie H., Shang Y. (2020). Effects of 16-form wheelchair Tai Chi on the autonomic nervous system among patients with spinal cord injury. *Journal of Alternative & Complementary Medicine*.

[48] Liberati G., Mulders D., Algoet M. (2020). Insular responses to transient painful and non-painful thermal and mechanical spinothalamic stimuli recorded using intracerebral EEG. *Scientific Reports*.

[49] Noble L. J., Meruva V. B., Hays S. A., Rennaker R. L., Kilgard M. P., McIntyre C. K. (2019). Vagus nerve stimulation promotes generalization of conditioned fear extinction and reduces anxiety in rats. *Brain Stimulation*.

[50] Zhang G. Q., Zhang W. (2009). Heart rate, lifespan, and mortality risk. *Ageing Research Reviews*.

[51] Pires F. O., Noakes T. D., Lima-Silva A. E. (2011). Cardiopulmonary, blood metabolite and rating of perceived exertion responses to constant exercises performed at different intensities until exhaustion. *British Journal of Sports Medicine*.

[52] Al-Mallah M. H., Juraschek S. P., Whelton S. (2016). Sex differences in cardiorespiratory fitness and all-cause mortality. *Mayo Clinic Proceedings*.

[53] Taylor L. E., Ramirez L. A., Musall J. B., Sullivan J. C. (2019). Tipping the scales: are females more at risk for obesity‐ and high‐fat diet‐induced hypertension and vascular dysfunction?. *British Journal of Pharmacology*.

[54] Jabbour G., Iancu H.-D. (2015). Mechanical efficiency improvement in relation to metabolic changes in sedentary obese adults. *BMJ Open Sport & Exercise Medicine*.

[55] Loe H., Rognmo Ø., Saltin B., Wisløff U. (2013). Aerobic capacity reference data in 3816 healthy men and women 20-90 years. *PLoS One*.

[56] Irzaldy A., Wiyasihati S. I., Purwanto B. (2016). Lung vital capacity of choir singers and nonsingers: a comparative study. *Journal of Voice*.

[57] Shrestha R., Shrestha A. P., Sonnenberg T., Mistry J., Shrestha R., MacKinney T. (2021). Needs assessment and identification of the multifaceted COPD care bundle in the emergency department of a tertiary hospital in Nepal. *International Journal of Chronic Obstructive Pulmonary Disease*.

[58] Baraniuk J. N., Jamieson M. J. (2010). Rhinorrhea, cough and fatigue in patients taking sitagliptin. *Allergy, Asthma & Clinical Immunology: Official Journal of the Canadian Society of Allergy and Clinical Immunology*.

[59] Fu S., Yin L., Lin X., Lu J., Wang X. (2018). Effects of cyclic mechanical stretch on the proliferation of L6 myoblasts and its mechanisms: PI3K/Akt and MAPK signal pathways regulated by IGF-1 receptor. *International Journal of Molecular Sciences*.

[60] Marin D. P., Macedo dos Santos R. D. C., Bolin A. P., Guerra B. A., Hatanaka E., Otton R. (2011). Cytokines and oxidative stress status following a handball game in elite male players. *Oxidative Medicine and Cellular Longevity*.

